# Prognostic significance of the aberrant expression of neuroendocrine markers in melanomas

**DOI:** 10.1186/s13000-021-01135-x

**Published:** 2021-08-28

**Authors:** Yan Wu, Yumei Lai, Miao Zhang, Zhongwu Li

**Affiliations:** Department of Pathology, Key Laboratory of Carcinogenesis and Translational Research (Ministry of Education), Peking University Cancer Hospital and Institute, No. 52, Fucheng Road, Haidian District, Beijing, 100142 China

**Keywords:** Melanoma, Neuroendocrine marker, Immunohistochemistry, Prognosis

## Abstract

**Background:**

Melanoma is a highly malignant tumor with diverse histopathological morphology and frequent aberrant expression of immunohistochemical markers. An occasionally reported phenomenon is the abnormal expression of neuroendocrine markers. Awareness of this situation is essential because such tumors need to be differentiated from neuroendocrine tumors because of their significant therapeutic and prognostic implications.

**Methods:**

We retrospectively analyzed the expression of chromogranin A (CgA), synaptophysin (Syn) and CD56 as neuroendocrine markers in 308 cases with melanomas. Kaplan-Meier curves and Cox regression analyses were used for overall survival (OS) and progression-free survival (PFS) evaluation and comparison between neuroendocrine markers expression status in all melanoma cases or stage I–II cases.

**Results:**

The expression of neuroendocrine markers in melanomas is not uncommon. CgA was positive in 6/304 (2.0%) cases, Syn in 26/304 (8.6%), and CD56 in 56/189 (29.6%). None of the cases co-expressed all the three markers. Focal or weak expression of at least one neuroendocrine marker was identified in 70/188 (37.2%) cases. The expression of CgA was correlated with age (*p* = 0.019), while the positive expression of Syn and CD56 showed borderline significance (*p* = 0.078 and 0.083, respectively), but not for any neuroendocrine marker expression. The expression of any neuroendocrine marker showed borderline significance with staging (*p* = 0.066). The expression of CgA, Syn, CD56, or any neuroendocrine marker did not correlate with clinicopathological features including sex, specimen type, origin, location, and histology subtype. Survival analyses revealed that the expression of neuroendocrine markers was not associated with OS or PFS.

**Conclusions:**

Our study confirms that neuroendocrine marker expression is a common phenomenon in melanomas, but it has no prognostic significance. Nevertheless, awareness can avoid misdiagnosis in cases of melanomas with unusual morphology and immunophenotypes.

## Background

Melanoma is a type of malignant tumor, which has a relatively good prognosis in the early stage; however, it becomes life-threatening at an advanced stage [1]. Therefore, timely and accurate diagnosis ensures active treatment of this disease. Pathology is the gold standard for melanoma diagnosis and mostly relying on histomorphology, along with clinical history and ancillary immunohistochemistry. Immunohistochemistry, however, is a double-edged sword that aids in the diagnosis of melanoma, but occasionally causes a potential diagnostic pitfall.

Aberrant immunohistochemical expression of melanoma has been previously reported [2]. HMB-45, Melan-A, S-100 and SOX10 are the most utilized markers for melanoma, while loss of expression of one or more melanocytic markers is not infrequent in primary and metastatic cases [3, 4]. S-100 has always been regarded as the most sensitive marker for melanoma, with 96–99% positivity, except for sinonasal melanoma [5, 6]. Conversely, aberrant expression of markers, other than melanocytic ones, represents the so-called metaplastic change in melanoma, including schwannian, smooth muscle [7], rhabdomyoblastic [8] and osteocartilaginous differentiation [9].

Although abnormal expression of neuroendocrine markers in melanomas has been reported occasionally, most were case reports and small case series [10, 11]. This situation has made the diagnosis quite a conundrum, especially in atypical or metastatic melanoma cases, which may be misdiagnosed as neuroendocrine tumors [12]. To the best of our knowledge, a larger case series was reported by Romano et al., without further referral to the prognostic significance of this phenomenon [5]. Currently, aberrant or anomalous expression of neuroendocrine markers has been described in a variety of tumors, including endometrial carcinoma, alveolar rhabdomyosarcoma, colorectal cancer, and prostate adenocarcinoma [13–15], and sometimes also coupled to specific clinical characteristics and worse prognosis [16]; however, their clinical significance in melanomas is uncertain.

Because of the small case series reported in the literature and the lack of correlation with prognosis, we retrospectively examined the expression of the three neuroendocrine markers, CgA, Syn, and CD56, in a large case series of 308 melanoma cases and its correlation with clinicopathological parameters and possible prognostic significance.

## Methods

### Patients and specimens

In a retrospective review of archived pathology files at Peking University Cancer Hospital and Institute in China, 308 melanoma specimens were diagnosed between 2000 and 2020. Previous definitive diagnoses of all cases were based on clinical history, histopathological evaluation, and immunohistochemistry staining, while some cases were diagnosed with the aid of molecular testing. The clinical information and molecular pathology test results of all enrolled patients were obtained by reviewing the electronic medical records. Follow-up information was obtained from medical records or by phone communication. Overall survival (OS) was calculated from the time of melanoma diagnosis to the time of death or the last follow-up. The duration of progression-free survival (PFS) was calculated from the date of surgery to the date of recurrence or progression. The present study was approved by the Ethics Committee of Peking University Cancer Hospital and Institute, and written consent was obtained from all patients.

The morphological subtype was determined by reviewing the stored electronic images independently by two pathologists (Wu Y and Lai Y). The clinical staging for melanoma was defined as I–II for tumors confined to the primary location, III for regional lymph node metastasis, and IV for distant metastases [17].

### Immunohistochemistry

Initially, 4-μm sections were cut from each formalin-fixed and paraffin-embedded melanoma block. Sequential procedures of dewaxed and rehydrated tissue sections were performed, followed by heat-mediated epitope retrieval with 10 mmol/L citrate buffer (pH 6.0). Endogenous peroxidase was inactivated using 3% hydrogen peroxide solution. The sections were incubated with a primary antibody working solution of CgA/Melan-A /CK/EMA/Cam5.2/Vimentin (Zhongshan Company, Beijing, China), Syn/S-100/SOX10 (GeneTex, Inc. Irvine, CA, USA), CD117 (Roche, Basel, Switzerland), and CD56/HMB45 (XiYa Reagent, Chengdu, China) at room temperature for 1 h. Not all markers could be detected as most of the enrolled cases came for consultation, and the immunohistochemical items detected were limited by the number of sections. The intensity score was defined using the following criteria: 0, no staining; 1, weak or partial staining; and 2, strong and diffuse staining.

### Statistics analyses

Measurement data are described as median and ranges. The correlation between categorical variables was calculated using Pearson’s chi-square or Fisher’s test. Kaplan-Meier survival curve was used to perform survival analysis, and the log-rank test was applied to evaluate statistical significance. Univariate and multivariate Cox proportional-hazards regression models were used to investigate the association between OS/DFS and clinical and pathological features. *P* < 0.05 indicates a statistically significant difference. All statistical analyses were performed using SPSS, version 22.0 (Chicago, IL, USA).

## Results

### Clinical and pathological characteristics

The clinical and pathological characteristics of all patients with melanoma are summarized in Table 1. Patients’ median age was 55.2 (7–87) years, and 170 (55.2%) were females. The sample cohort included 84 (27.3%) biopsy samples and 224 (72.7%) excision specimens. The primary anatomic sites included the skin (33, 10.7%), mucosa (176, 57.1%), acral area (30, 9.7%), eye (8, 2.6%), meninges (1, 0.3%), and unknown primary origin (60, 19.5%). A total of 192 (62.3%) specimens were of primary origin, 112 (36.4%) had distant recurrence, and 4 (1.3%) had local recurrence. Histological subtypes were retrospectively analyzed in 276 cases with melanomas, 128 (46.3%) of which were of epithelioid type, 17 (6.2%) were of the spindle type, 96 (34.8%) were of the small round cell type, 10 (3.6%) were of the pleomorphic type, and 25 (9.1%) were of the mix type. At the initial diagnosis, 139 (46.8%) were diagnosed as stage I–II, 39 (13.1%) as stage III, and 119 (40.1%) as stage IV. Among 195 cases with molecular test results, 41 (21.0%) harbored BRAF alternations, of which 37 (90.2%) cases exhibited a V600E mutation, while other rare molecular alternations included 1 G469A, 1 G466E, 1 BRAF rearrangement (BRAF-CADPS2 and BRAF-exOC4), and 1 c.95_100del. KIT mutations were detected in 12 (6.2%) melanoma patients, with the most frequent mutations being L576P, N822K, and R634Q. 23 (11.8%) specimens contained mutations in the NRAS gene, including 12 Q61R, followed by Q61k and Q61H. Only one (0.5%) patient had a PDGFRA Y849C mutation.

**Table 1:** Clinicopathological features of 308 patients with melanoma

Clinicopathologic features	No. (%)
Age at surgery (years)	55.2 (7–87)
Sex	
Female	170 (55.2)
Male	138 (44.8)
Specimen type	
Biopsy	84 (27.3)
Excision	224 (72.7)
Location	
Skin	33 (10.7)
Mucosa	176 (57.1)
Acral	30 (9.7)
Eye	8 (2.6)
Other	61 (19.8)
Origin	
Primary	192 (62.3)
Distant recurrence	112 (36.4)
Local recurrence	4 (1.3)
Histological subtype	
Epithelioid	128 (46.3)
Spindle cell	17 (6.2)
Small round cell	96 (34.8)
Pleomorphic	10 (3.6)
Mix type	25 (9.1)
Molecular alternations	
BRAF	41 (21.0)
KIT	12 (6.2)
PDGFRA	1 (0.5)
NRAS	23 (11.8)
Stage	
I–II	139 (46.8)
III	39 (13.1)
IV	119 (40.1)
Overall survival (months)	16 (0–233)
Progression-free survival (months)	10 (1–225)

The OS ranged from 0 to 233 months, with a median duration of 16 months. Of the 266 melanoma patients, 126 were alive at the last follow-up, and the 1-, 3-, and 5-year survival rates were 73.3, 41.6, and 30.7%, respectively. In 150 melanoma patients, disease progression occurred in 132 cases, and PFS ranged from 1 to 225 months with a median duration of 10 months. At the last follow-up, 6 patients were lost to follow-up. Of the remaining 144 patients, 18 (12.5%) remained alive without evidence of progression, 68 (47.2%) were alive with disease progression, and 58 (40.3%) died of the disease.

### Immunostaining and clinicopathological features

**Fig. 1:**
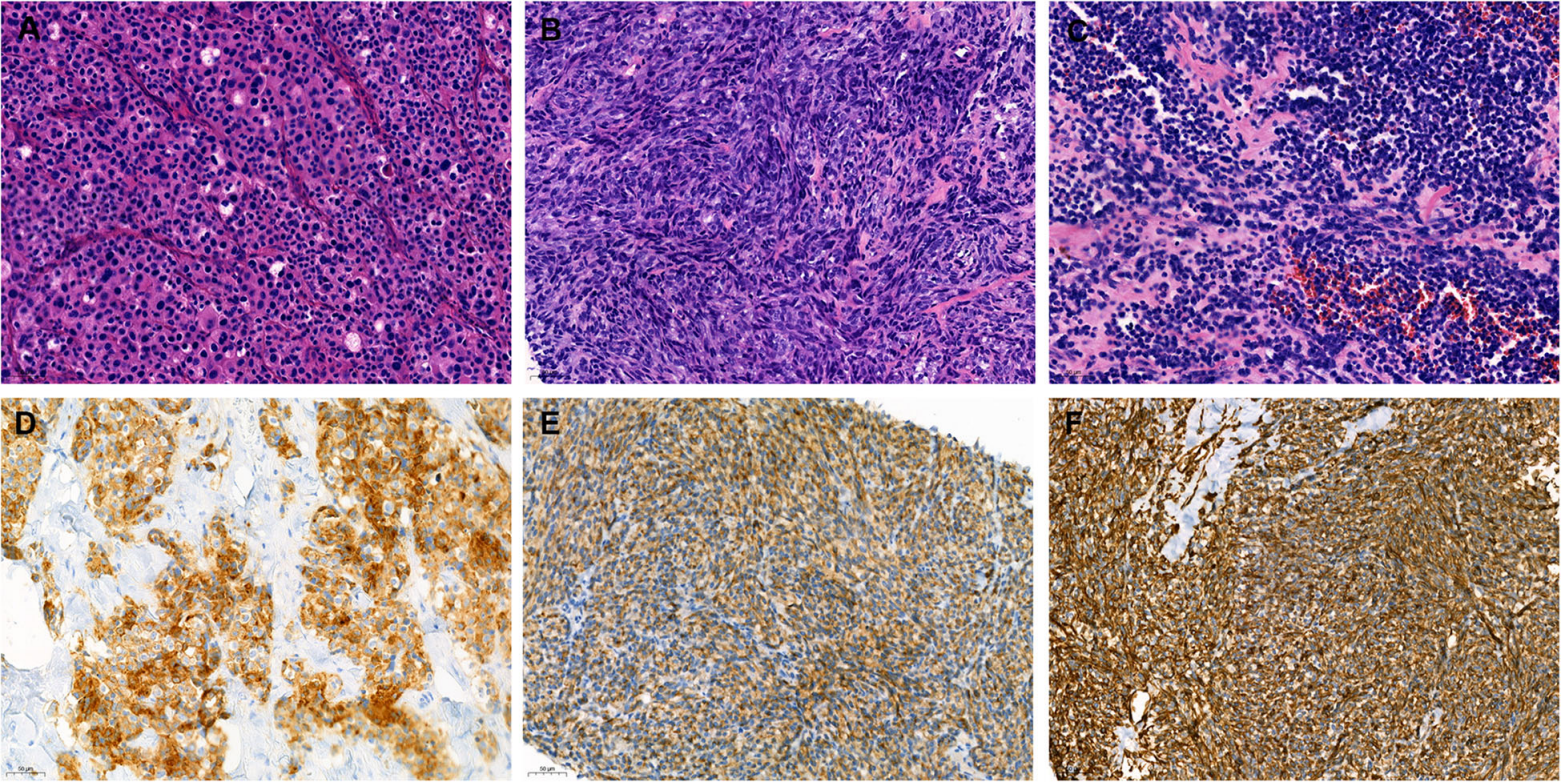
Representative images of H&E staining (**A**-**C**) and immunohistochemical staining (**D**-**F**) in melanomas. **A** epithelioid subtype, **B** spindle cell subtype, **C** small round cell type, **D** Syn, **E** CD56, **F** Vimentin (magnification: ×200)

In our series, CgA was only positive in 6/304 (2.0%) cases, all of which were weakly or focally positive. Syn was positive in 26/304 (8.6%) cases, of which 8 stained diffuse and strongly positive, and 18 stained weakly or focally positive. By comparison, CD56 had a higher positive expression rate in 56/189 (29.6%) cases, of which 35 were diffuse and strongly positive, and 21 were weakly positive. Of the 188 cases in which CgA, Syn and CD56 were detected simultaneously, focal or weak expression of at least one of the studied markers was observed in 70/188 (37.2%) cases (Table 2). Representative images of H&E staining and immunolabeling are shown in Fig. 1.

**Table 2:** Immunohistochemical findings

Antigen	Total
CgA	6/304 (2.0%)
Syn	26/304 (8.6%)
CD56	56/189 (29.6%)
Neuroendocrine^a^	70/188 (37.2%)
HMB-45	235/264 (89.0%)
Melan-A	199/231 (86.1%)
S−100	224/254 (88.2%)
SOX10	50/55 (90.9%)
CD117	27/39 (69.2%)
CK	6/242 (2.5%)
EMA	2/63 (3.2%)
Cam5.2	1/49 (2.0%)
Vimentin	148/151 (98.0%)

^a^ CgA, Syn or CD56

Associations between neuroendocrine marker expression and clinicopathological features are summarized in Table 3. CgA expression was correlated with age (*p* = 0.019), while the positive expression of Syn and CD56 showed borderline significance with age (*p* = 0.078 and 0.083, respectively), whereas no correlation was observed between neuroendocrine marker expression and age (*p* = 0.463). CgA, Syn, or CD56 expression did not correlate with staging, but neuroendocrine marker positive expression showed a borderline correlation with staging (*p* = 0.066). Patients with stage I–II and stage III had similar positive rates, i.e., 34/85 (40.0%) and 12/27 (44.4%), respectively, but it declined to 18/68 (26.5%) in stage IV patients. Sex, specimen type, origin, location, and histological subtype were not related to neuroendocrine marker expression.

**Table 3:** Correlation between neuroendocrine marker expression and clinicopathological features in melanoma patients

Clinicopathologic features	CgA (%)	p	Syn (%)	p	CD56 (%)	p	Neuroendocrine^a^ (%)	p
Age (years)								
≤ 55	6/154 (3.9)	0.019*	18/154 (11.7)	0.078	21/92 (22.8)	0.083	32/91 (35.2)	0.463
>55	0/150 (0.0)		8/150 (5.3)		35/97 (36.1)		38/97 (39.2)	
Sex								
Female	3/169 (1.8)	0.539	14/168 (8.3)	1.000	34/112 (30.4)	0.190	44/111 (39.6)	0.170
Male	3/135 (2.2)		12/136 (8.8)		22/77 (28.6)		26/77 (33.8)	
Specimen type								
Excision	5/222 (2.3)	0.546	16/221 (7.2)	0.353	42/137 (30.7)	0.875	49/136 (36.0)	0.845
Biopsy	1/82 (1.2)		10/83 (12.0)		14/52 (26.9)		21/52 (40.4)	
Origin								
Primary	4/190 (2.1)	0.893	14/190 (7.4)	0.650	36/112 (32.1)	0.259	42/111 (37.8)	0.304
Distant recurrence	2/110 (1.8)		12/110 (10.9)		19/73 (26.0)		27/73 (40.0)	
Local recurrence	0/4 (0)		0/4 (0)		1/4 (25.0)		1/3 (33.3)	
Location								
Skin	0/32 (0.0)	0.147	7/32 (21.9)	0.069	6/23 (26.1)	0.128	9/23 (39.1)	0.309
Mucosa	4/175 (2.3)		12/175 (6.9)		40/106 (37.7)		45/105 (42.9)	
Acral	2/30 (6.7)		2/30 (6.7)		4/19 (21.1)		7/19 (36.8)	
Other	0/67 (0.0)		5/67 (7.5)		6/41 (14.6)		9/41 (22.0)	
Histology subtype								
Epithelioid	2/126 (1.6)	0.272	13/126 (10.3)	0.142	23/77 (29.9)	0.390	29/77 (37.7)	0.662
Spindle	0/17 (0.0)		1/17 (5.9)		1/8 (12.5)		2/8 (25.0)	
Small round cell	2/96 (2.1)		5/95 (5.3)		24/68 (35.3)		28/67 (41.8)	
Polymorphic	0/10 (0.0)		0/10 (0.0)		1/5 (20.0)		1/5 (20.0)	
Mix type	0/23 (0.0)		0/24 (0.0)		4/13 (30.8)		4/13 (30.8)	
Stage								
I-II	4/136 (2.9)	0.872	11/136 (8.1)	0.106	27/86 (31.4)	0.109	34/85 (40.0)	0.066
III	0/39 (0.0)		8/39 (20.5)		10/27 (37.0)		12/27 (44.4)	
IV	2/118 (1.7)		6/118 (5.1)		14/68 (20.6)		18/68 (26.5)	

^a^ CgA, Syn or CD56

* *p* < 0.05

### Survival analyses

**Fig. 2:**
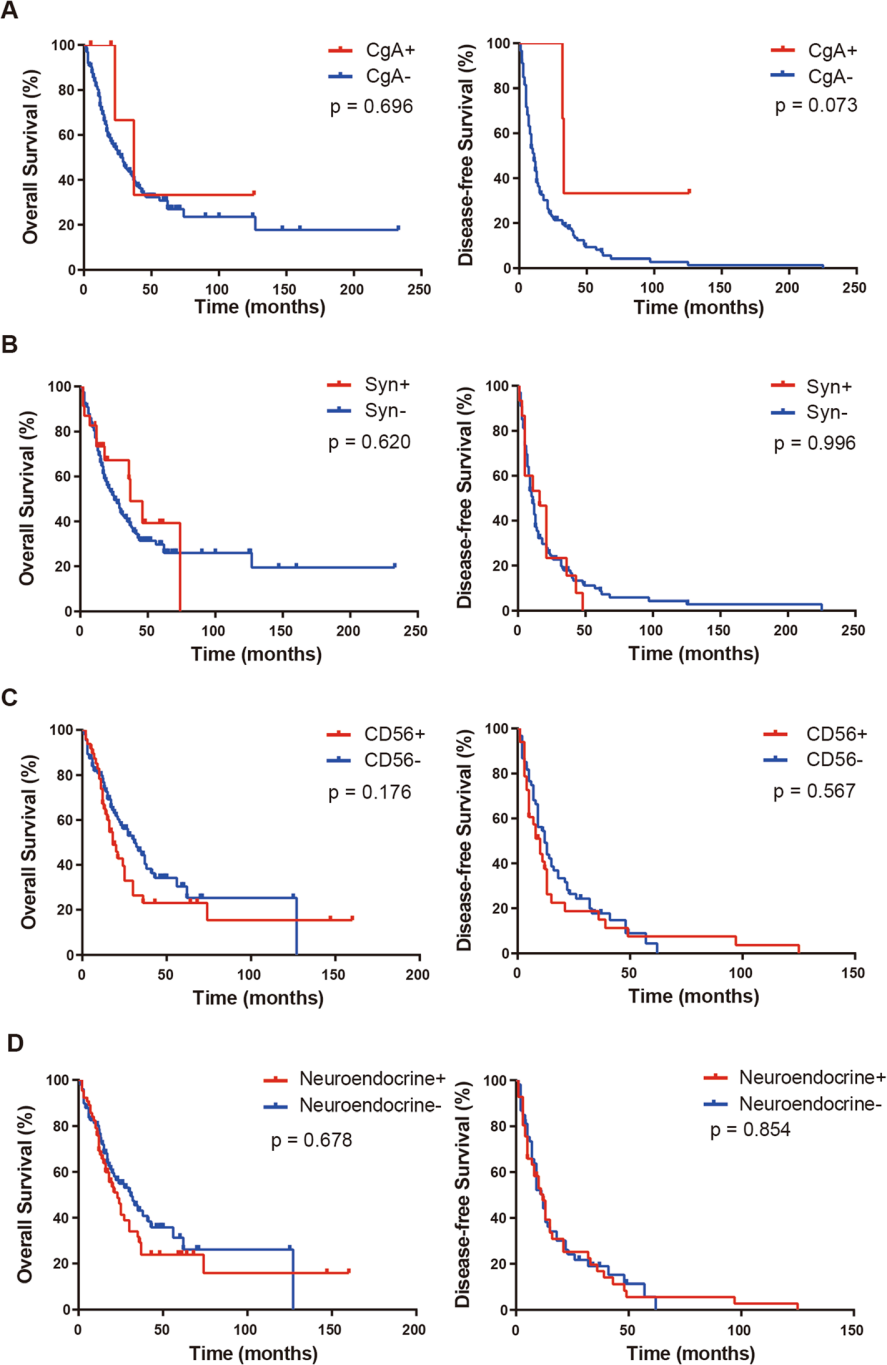
Kaplan-Meier survival analysis of melanoma patients based on expression status of CgA, Syn, CD56 and any neuroendocrine marker

Kaplan-Meier survival analysis indicated that the OS and DFS between melanoma patients with positive and negative neuroendocrine expression did not show statistically significant differences (Fig. 2). Associations of the features studied with the OS and PFS are summarized in Tables 4, 5, 6 and 7. Similarly, in univariate and multivariable analyses, the expression of CgA, Syn, CD56 or any neuroendocrine marker had no effect on OS and PFS. In univariate and multivariable analyses, female melanoma patients had a significantly higher OS (hazard ratio [HR] = 0.613, *p* = 0.072; HR = 0.548, *p* = 0.039, respectively), while stage IV patients had a significantly lower OS (HR = 4.456, *p* = 0.000; HR = 4.194, p = 0.000, respectively). In univariate analysis, patients whose primary site was located in the mucosa or others (eye, meninges and unknown primary origin) had shorter OS than those whose primary site was located in the skin (HR = 2.611, *p* = 0.026; HR = 3.222, *p* = 0.017, respectively), and patients with BRAF mutation had longer OS than those with the wild type (HR = 0.440, *p* = 0.031). Nevertheless, in multivariate analysis, primary location and BRAF mutation status had no influence on OS. Mucosal melanoma was associated with an increased risk of tumor progression after surgery in univariate and multivariate analyses (HR = 2.636, *p* = 0.013; HR = 2.644, *p* = 0.032, respectively). Sex (female) showed borderline significance with PFS (HR = 0.612, *p* = 0.074; HR = 0.552, *p* = 0.056, respectively). Age (≤55 years) and primary location in the mucosa of stage I–II melanoma patients were significantly correlated with OS in univariate analysis (HR = 0.413, *p* = 0.038; HR = 3.246, *p* = 0.044, respectively) but not in multivariate analysis. Female sex was an independent factor that predicted longer OS in stage I–II melanoma patients in the multivariate analysis (HR = 0.326, *p* = 0.019). In the PFS analysis of stage I–II melanoma patients, univariate and multivariate analyses revealed that mucosal location was associated with shorter PFS duration (HR = 2.636, *p* = 0.013; HR = 2.667, *p* = 0.032, respectively).

**Table 4:** Univariate and multivariable Cox proportional hazards model for OS

Characteristic	Univariate analysis		Univariate analysis	
	HR (95% CI)	p	HR (95% CI)	p
Age				
(≤55 vs. >55)	1.142 (0.679–1.922)	0.617	0.696 (0.390–1.243)	0.221
Sex				
Female vs. Male	0.613 (0.360–1.044)	0.072	0.548 (0.309–0.970)	0.039*
Location				
Mucosa vs. Skin	2.611 (1.123–6.070)	0.026*	2.046 (0.834–5.020)	0.118
Acral vs. Skin	1.573 (0.497–4.974)	0.441	1.503 (0.441–5.125)	0.515
Others vs. Skin	3.222 (1.235–8.407)	0.017*	1.933 (0.705–5.304)	0.200
Neuroendocrine^a^	1.067 (0.632–1.802)	0.809	1.323 (0.767–2.282)	0.314
BRAF alternations	0.440 (0.208–0.930)	0.031*	0.608 (0.262–1.412)	0.247
Stage				
III vs. I-II	0.787 (0.297–2.082)	0.629	0.617 (0.222–1.718)	0.355
IV vs. I-II	4.456 (2.500–7.940)	0.000***	4.194 (2.185–8.050)	0.000***

^a^ CgA, Syn or CD56

* *p* < 0.05; *** *p* < 0.005

**Table 5:** Univariate and multivariable Cox proportional hazards model for PFS

Characteristic	Univariate analysis		Multivariable analysis	
	HR (95% CI)	p	HR (95% CI)	p
Age				
≤ 55 vs. >55	0.823 (0.490–1.382)	0.462	0.783 (0.456–1.343)	0.374
Sex				
Female vs. Male	0.612 (0.357–1.049)	0.074	0.552 (0.301–1.016)	0.056
Location				
Mucosa vs. Skin	2.636 (1.230–5.680)	0.013*	2.644 (1.085–6.442)	0.032*
Acral vs. Skin	2.064 (0.823–5.173)	0.122	2.018 (0.723–5.630)	0.180
Other vs. Skin	1.186 (0.395–3.558)	0.761	0.844 (0.460–1.549)	0.584
Neuroendocrine^a^	0.854 (0.510–1.432)	0.550	0.844 (0.460–1.549)	0.584
BRAF alternations	0.797 (0.452–1.406)	0.434	1.035 (0.517–2.069)	0.923
Stage				
III vs. I-II	1.084 (0.591–1.986)	0.795	1.197 (0.601–2.384)	0.610
IV vs. I-II	0.946 (0.291–3.076)	0.927	0.919 (0.267–3.160)	0.893

^a^ CgA, Syn or CD56

* *p* < 0.05

**Table 6:** Univariate and multivariable Cox proportional hazards model for OS in stage I-II melanoma patients

Characteristic	Univariate analysis		Multivariable analysis	
	HR (95% CI)	p	HR (95% CI)	p
Age				
≤ 55 vs.>55	0.413 (0.179–0.953)	0.038*	0.951 (0.407–2.221)	0.908
Sex				
Female vs. Male	1.110 (0.499–2.471)	0.797	0.326 (0.128–0.833)	0.019*
Location				
Mucosa vs. Skin	3.246 (1.031–10.225)	0.044*	1.619 (0.386–6.781)	0.510
Acral vs. Skin	1.242 (0.227–6.800)	0.803	0.736 (0.102–5.324)	0.736
Neuroendocrine^a^	0.933 (0.412–2.112)	0.868	0.775 (0.305–1.971)	0.593
BRAF alternations	0.330 (0.098–1.105)	0.072	0.304 (0.065–1.417)	0.129

^a^ CgA, Syn or CD56

* *p* < 0.05

**Table 7:** Univariate and multivariable Cox proportional hazards model for PFS in stage I-II melanoma patients

Characteristic	Univariate analysis		Multivariable analysis	
	HR (95% CI)	p	HR (95% CI)	p
Age				
≤ 55 vs. >55	0.612 (0.357–1.049)	0.074	0.770 (0.451–1.315)	0.338
Sex				
Female vs. Male	0.823 (0.490–1.382)	0.462	0.542 (0.298–0.988)	0.045*
Location				
Mucosa vs. Skin	2.636 (1.223–5.680)	0.013*	2.667 (1.090–6.526)	0.032*
Acral vs. Skin	2.064 (0.823–5.173)	0.122	2.113 (0.767–5.824)	0.148
Other vs. Skin	1.186 (0.395–3.558)	0.761	1.069 (0.321–3.560)	0.913
Neuroendocrine^a^	0.854 (0.510–1.432)	0.550	0.865 (0.477–1.569)	0.633
BRAF alternations	0.797 (0.452–1.406)	0.434	1.071 (0.546–2.101)	0.842

^a^ CgA, Syn or CD56

* *p* < 0.05

## Discussion

Melanoma has diverse histopathological morphologies, including loss of melanin, partial or complete loss of melanoma markers and aberrant expression of non-melanocytic markers, representing potentially diagnostic pitfalls [18]. Accurate diagnosis of melanoma is of great clinical importance because of its different biological behavior and therapeutic approach.

CgA, Syn, and CD56 are the most frequently used markers of neuroendocrine differentiation, but the expression of one or more of these markers may also be found in a subset of non-neuroendocrine tumors that may obscure the correct diagnosis [15]. Aberrant neuroendocrine marker expression in melanomas seems to be a diagnostic conundrum in daily practice, especially in metastatic sites and tissue biopsies. To investigate the expression frequency of CgA, Syn, and CD56, we enrolled 308 melanoma specimens, including 84 (27.3%) biopsies. In this study, CgA was positive in 6/304 (2.0%), Syn in 26/304 (8.6%), and CD56 in 56/189 (29.6%) cases; however, these markers were not simultaneously expressed in any case. Overall, melanoma with any neuroendocrine marker expression was observed in 70/188 (37.2%) of patients. This finding is in concordance with the finding of a previous report by Steppert et al. that the positive expression of CD56 was most frequently expressed compared with that of CgA and Syn [12]. The results prompted us to conclude that the abnormal expression of neuroendocrine markers is a relatively frequent event in melanoma, and careful interpretation of these markers is of critical importance. Because of the lack of sample volume, accurate diagnosis of melanoma in the core-needle biopsy is more intractable, especially in a morphologically atypical case with aberrant positivity for neuroendocrine markers. The differential diagnosis for melanoma expressing neuroendocrine markers includes neuroendocrine carcinoma (NEC) and metastatic Merkel cell carcinoma (MCC). The latter two are commonly seen in older adults and express epithelial markers panCK and high Ki67 index but do not express melanocytic markers. MCC is also positive for CK20 and sometimes CK7. Occasionally, loss of melanocytic marker expression is associated with poor differentiation; thereby, the patient’s clinical history should be taken into consideration. Ultrastructural examination of melanoma revealed the existence of neuroendocrine granules associated with melanosomes in tumor cells, suggesting definite neuroendocrine differentiation [19, 20].

Neuroendocrine differentiation in melanoma has been observed, but its prognostic significance is uncertain [21]. The results of this study demonstrated that, the expression of neuroendocrine markers in melanoma did not significantly impact the OS and PFS. This is quite different from other tumors. Not an uncommon event in primary colorectal cancer, endocrine differentiation could serve as a negative prognostic marker [22]. Although the results of prostate cancer with focal neuroendocrine differentiation were inconsistent, a recent meta-analysis showed that focal neuroendocrine differentiation is associated with worse prognosis [15].

It is widely acknowledged that CD56 is a sensitive but non-specific marker of neuroendocrine tumors in comparison to Syn and CgA [23]. Aberrant CD56 expression has been reported in basaloid anal squamous cell carcinoma [24], pulmonary adenocarcinoma and squamous cell carcinoma [25], and adrenocortical carcinoma [26]. Therefore, the diagnosis of a poorly differentiated tumor as neuroendocrine cancer using a single marker, CD56, must be made carefully. Melanocytes originate from the neural crest cells and migrate to all body parts [27], which share a common ancestry with neuroendocrine cells from the neuroectoderm, which may partially explain the phenomenon of melanomas expressing neuroendocrine markers. In our series, no significant difference was observed in the positive rate of neuroendocrine markers among different histological subtypes of melanoma, which is consistent with that reported by other studies [28]. This is quite different from the digestive tract, as histological structural pattern and cell morphology may indicate neuroendocrine differentiation.

One limitation of our study is that 290 cases enrolled in this series came for consultation, and the results of molecular detection and immunohistochemistry assays were derived from our routine practice. This selection bias, may have affected the results of this study.

## Conclusions

The expression of neuroendocrine markers seems to be common in melanoma; therefore, we should keep in mind that the cases with aberrant expression of these markers also have the possibility to be melanoma, which should be listed in the differential diagnosis. However, unlike other cancers, neuroendocrine marker expression in melanomas does not indicate a worse prognosis.

## Data Availability

Not applicable.

## Abbreviations

CgA: Chromogranin-A
Syn: Synaptophysin
OS: Overall survival
PFS: Progression-free survival
HR: Hazard ratio
CI: Confidence interval
